# Surgical resection of a middle mediastinal paraganglioma that caused diabetes

**DOI:** 10.1186/s40792-020-00983-x

**Published:** 2020-09-30

**Authors:** Kentaro Miura, Nobutaka Kobayashi, Hidetoshi Satomi

**Affiliations:** 1 Department of Thoracic Surgery, Japanese Red Cross Society Nagano Hospital, 5-22-1 Wakasato, Nagano, 380-8582 Japan; 2 Department of Pathology, Japanese Red Cross Society Nagano Hospital, Nagano, Japan

**Keywords:** Paraganglioma, Middle mediastinum, Functional, Surgical resection, Diabetes

## Abstract

**Background:**

Paragangliomas are rare neuroendocrine tumors originating from chromaffin cells of extra-adrenal origin. Ninety percent of adrenergic tumors originate in the adrenal medulla and are known as pheochromocytomas; the remaining 10% are extra-adrenal and are called paragangliomas. Mediastinum paragangliomas is rare and commonly originate from the posterior mediastinum, while those originating from the middle posterior are quite rare. Some paragangliomas secrete catecholamines, leading to symptoms such as hypertension, tachycardia, and diabetes.

**Case presentation:**

A 76-year-old woman visited our hospital for the treatment and further evaluation of diabetes. Her hemoglobin A1c levels had risen to 11.0%. To investigate the cause of her diabetes, a contrast-enhanced chest computed tomography scan was performed, revealing a ring-enhancing tumor (30 × 30 mm) in the middle mediastinum. The surgical resection was performed via video-assisted thoracic surgery. Surgery was performed using a vessel-sealing device; however, bleeding was persistent from the surrounding tissue. Total bleeding was 400 g. Blood pressure fluctuations and arrhythmia did not occur during the operation. The patient’s uncontrolled diabetes was cured after the surgery, and the tumor was diagnosed as a functional paraganglioma.

**Conclusions:**

We encountered a rare case of functional paraganglioma located in the middle mediastinum. Functional paragangliomas should be considered as a potential cause of uncontrolled diabetes, and a whole-body CT scan should be performed to investigate this possible cause.

## Background

Paragangliomas are rare neuroendocrine tumors originating from chromaffin cells of extra-adrenal origin. Ninety percent of adrenergic tumors originate in the adrenal medulla and are known as pheochromocytomas; the remaining 10% are extra-adrenal and are called paragangliomas [1]. Paragangliomas originating from the mediastinum account for 1–2% of all paragangliomas and less than 0.3% of all mediastinal tumors [2–4]. Mediastinum paragangliomas commonly originate from the posterior mediastinum, while those originating from the middle mediastinum are quite rare [1]. Between 50–80% of paragangliomas are non-functional; however, some paragangliomas secrete catecholamines, leading to symptoms such as hypertension, tachycardia, and diabetes [4]. Surgical resection is recommended; however, blood-pressure fluctuations and massive bleeding are concerns during surgery. We encountered a rare case of functional paraganglioma originating from the middle mediastinum, and the patient’s uncontrolled diabetes was cured after surgical resection.

## Case presentation

A 76-year-old woman visited our hospital for the treatment and further evaluation of diabetes. She had complained of malaise for several months and was diagnosed with diabetes in another hospital. She had a history of hyperlipidemia in addition to diabetes, and was treated with oral medication. Her blood tests were within the normal range except for her hemoglobin A1c levels, which had risen to 11.0%. Her performance status was normal, although she had malaise and anorexia. Her blood pressure was 114/72 mmHg and pulse was 80 beats/min. Her body weight was 54.4 kg. Urinary glucose was not detected. Her electrocardiogram was within normal limit. She had not been treated with adrenocorticotropic hormone (ACTH) and cortisol.

**Fig. 1:**
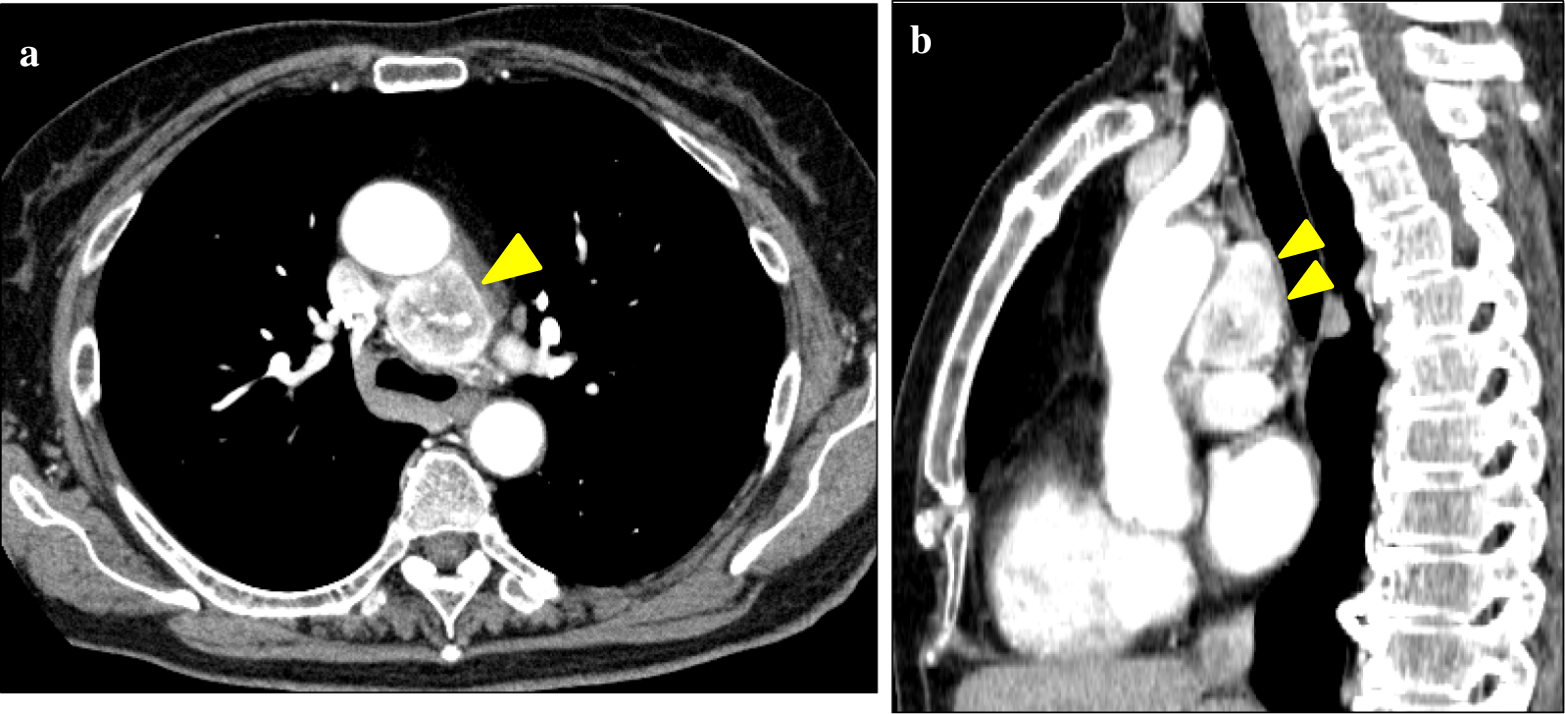
Chest CT scan. Preoperative contrast-enhanced chest computed tomography scan showing a ring-enhancing tumor in the middle mediastinum (allow)

A contrast-enhanced chest computed tomography (CT) scan was performed for the whole-body examinations, and revealed a ring-enhancing tumor (30 × 30 mm) in the middle mediastinum (Fig. 1). An endobronchial ultrasound-guided transbronchial needle aspiration (EBUS-TBNA) was also performed; however, it proved non-diagnostic.

Surgical resection was performed using a left-sided video-assisted thoracic surgery (VATS) approach. The tumor was located between the aorta and the left pulmonary artery. The surgery was performed using a vessel-sealing device; however, bleeding was persistent from the surrounding tissue. Total bleeding was 400 g. Blood pressure fluctuations and arrhythmia did not occur during the procedure, and the tumor was completely resected. Systolic blood pressure remained around 120, although it temporarily rose to 160 immediately after the start of the surgery. In addition, intraoperative blood sugar was stable, and it had been around 130 to 160. The postoperative course was generally good; however, the patient had hoarseness that seemed to be a left recurrent nerve palsy. She was discharged 6 days after surgery.

**Fig. 2:**
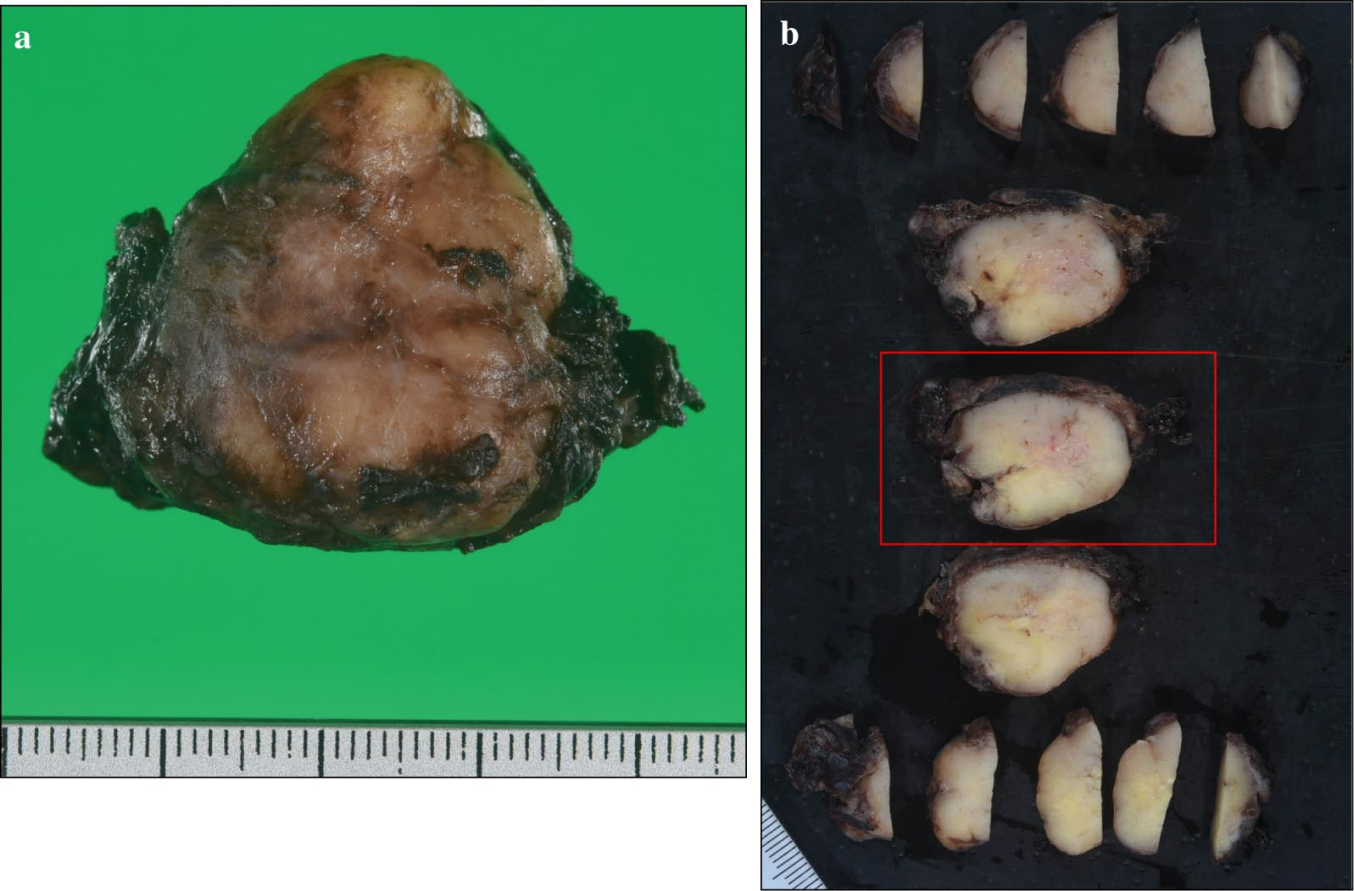
Macroscopic appearance. Tumor was 3.5 × 3.5 × 1.5 cm in diameter

**Fig. 3:**
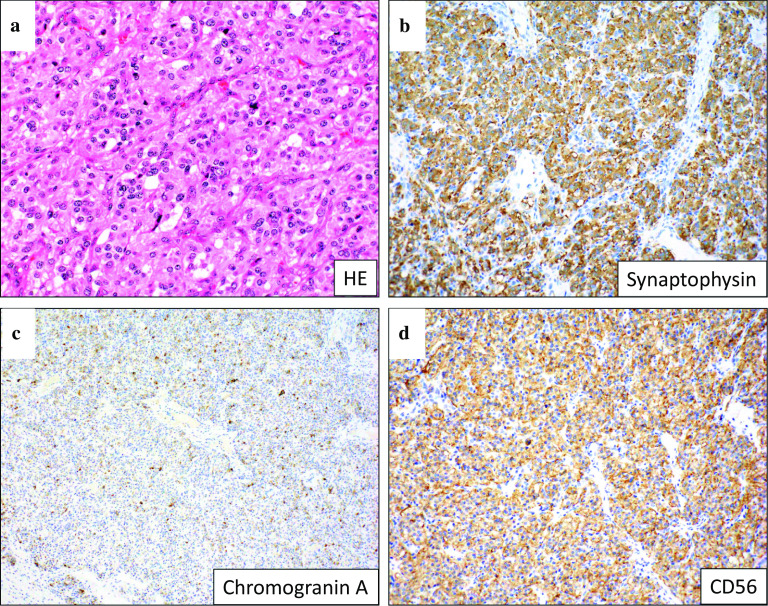
Microscopic appearance. **a** Hematoxylin–eosin staining showed that the tumor cells, with clear cytoplasm, were arranged in nests (×100). **b**–**d** The tumor was immunoreactive for synaptophysin, chromogranin A, and CD56 protein, and was diagnosed as a paraganglioma arising in the middle mediastinum (×100)

Histopathological examination of the tumor is shown, and it was 3.5 × 3.5 × 1.5 cm in diameter (Fig. 2a, b). Hematoxylin–eosin staining revealed that the tumor cells, with clear cytoplasm, were arranged in nests. The tumor was immunoreactive for synaptophysin, chromogranin A, and CD56 protein, and was diagnosed as a paraganglioma arising in the middle mediastinum (Fig. 3a–d). Ki-67-positive cells were 6–8%. The patient’s HbA1c levels improved to 5.6% in the 6 months after surgery, and all oral medications for diabetes were stopped. In addition, her appetite improved and her body weight recovered by 5 kg. The tumor was considered a functional paraganglioma.

## Discussion

The present case illustrates some important findings. First, the patient’s uncontrolled diabetes was completely cured after resecting a functional paraganglioma originating from the middle mediastinum.

Beninato et al. reported the outcomes of 153 patients with pheochromocytoma who underwent surgical resection, and diabetes was observed in 36 (23.4%) patients [5]. Overall, 93% of these patients showed improvement in their diabetes after surgical resection. There are no large-scale studies that refer to paraganglioma alone; however, if complete resection is performed, diabetes can likely be cured. Like the present case, a whole-body examination may be needed, because paragangliomas are a possible cause of uncontrolled diabetes.

Paragangliomas originating from the middle mediastinum are rare, but they should be considered if a strongly contrasted tumor is detected in a contrast-enhanced chest CT scan. It is recommended that measurements of plasma-free metanephrines or urinary fractionated metanephrines be tested if a paraganglioma is suspected [6]. Lenders et al. indicated that plasma-free metanephrines provide the best test for excluding or confirming pheochromocytoma, and its sensitivity was 99% and specificity was 89% [7]. In the current case, these metanephrines were not examined before surgery; however, a mediastinum paraganglioma should be listed as a differential diagnosis and these metanephrines should be examined before surgical resection. Almost all paragangliomas are benign, but they can recur, and lifelong follow-up is, therefore, recommended to detect recurrence or metastatic disease [6]. In addition, genetic testing for succinate dehydrogenase mutations is recommended, because paragangliomas are potentially hereditary [6].

We consider that it is important to perform whole-body examinations in patients with uncontrolled diabetes, because functional paraganglioma may cause diabetes. Paraganglioma should be considered if a ring-enhancing solid tumor is detected, and biochemical testing, such as measurements of plasma-free metanephrines or urinary fractionated metanephrines, should be performed.

Some reports indicate that a biopsy, such as EBUS-TBNA, before a surgical resection is dangerous because of the risk of bleeding or of blood-pressure alterations [8, 9]. Thus, complete surgical resection should first be considered, without a definitive diagnosis before the surgery. Because a large amount of bleeding is expected, a thoracotomy can be used instead of a VATS approach. In addition, preoperative vascular embolization may also be effective. Either way, ample, preoperative preparation should be performed. Attention should be paid to intraoperative blood-pressure fluctuations caused by intraoperative catecholamine secretion. Surgical resection of middle mediastinal tumor is often difficult to keep the good operative field, because it is surrounded by great vessels, pericardium, esophagus, or trachea, and careful attention should be paid to recurrent laryngeal nerve palsy.

## Conclusions

We encountered a case of functional paraganglioma located in the middle mediastinum, and after surgical resection, the patient’s uncontrolled diabetes was completely cured. Functional paragangliomas should, therefore, be considered as a potential cause of uncontrolled diabetes, and a whole-body CT scan should be performed to investigate this possible cause.

## Data Availability

The data used in this report are available from the corresponding author on reasonable request.
